# Effects of age on the genetic and clinical characteristics of retinitis pigmentosa

**DOI:** 10.3389/fopht.2026.1776570

**Published:** 2026-06-26

**Authors:** Yoshito Koyanagi, Sakurako Shimokawa, Takahiro Hisai, Kaho Yamamoto, Yasuhiro Ikeda, Koh-Hei Sonoda, Yusuke Murakami

**Affiliations:** 1 Department of Ophthalmology, Graduate School of Medical Sciences, Kyushu University, Fukuoka, Japan; 2 Department of Ophthalmology, Faculty of Medicine, University of Miyazaki, Miyazaki, Japan

**Keywords:** age, causative genes, genetic testing, onset, retinitis pigmentosa

## Abstract

**Purpose:**

This study aimed to investigate how age affects the genetic and clinical characteristics of retinitis pigmentosa (RP), focusing on the detectability of causative genes and the age at disease onset.

**Methods:**

We conducted a single-center study of 506 patients with RP who underwent comprehensive genetic testing through targeted resequencing of 83 known RP-associated genes using next-generation sequencing. Patients were stratified by age at study entry into six groups: <40 years (20–39), 40s (40–49), 50s (50–59), 60s (60–69), 70s (70–79), and ≥80 years. Detection rates of causative genes were calculated and compared across age groups using the Cochran-Armitage trend test. Genetically solved cases included 42 with *EYS*, 19 with *USH2A*, 9 with *RP1*, 14 with *RHO*, and 7 with *RPGR*. Clinical data were collected retrospectively. Age at onset was defined as the age when the patient first noticed night blindness, visual field constriction, or decreased best corrected visual acuity. Age at onset was compared across causative genes using an one-way analysis of variance (ANOVA). For pairwise comparisons, the Wilcoxon rank-sum test was applied with Bonferroni correction to adjust for multiple testing.

**Results:**

The mean age of participants was 58.8 years, and our sample included 235 males and 271 females. Case numbers by age group were as follows: <40 years, 58; 40s, 92; 50s, 94; 60s, 125; 70s, 104; and ≥80 years, 33. Detection rates of causative genes declined steadily with age: 39.7% (<40), 41.3% (40s), 36.2% (50s), 27.2% (60s), 19.2% (70s), and 3.0% (≥80), showing a statistically significant trend (p = 8.22 × 10^-7^, Cochran-Armitage trend test). In subset analysis, mean onset ages were *RPGR* (5.2 years), *EYS* (19.5 years), *RHO* (24.3 years), *RP1* (25.2 years), and *USH2A* (34.1 years), indicating a significant difference among genes (p < 0.001). Pairwise comparisons showed significantly earlier onset in the *RPGR* group relative to *USH2A* (p = 0.004).

**Conclusions:**

The detection rate of known causative genes of RP was lower in the elderly patients, potentially reflecting factors associated with a late-onset phenotype.

## 1 Introduction

Retinitis pigmentosa (RP), a major phenotype of inherited retinal dystrophies, is characterized by primary progressive degeneration from rod to cone cells, which typically results in night blindness, progressive constriction of the visual field, and central vision loss (1–4). More than 100 causative genes have been identified to date, and the advent of next-generation sequencing has substantially improved diagnostic detectability, enabling genetic counseling and enrollment in gene-targeted clinical trials (1–5).

Understanding how age modifies both the likelihood of genetic detection, and the timing of clinical onset is clinically relevant. Certain subtypes manifest during childhood or early adulthood, whereas others present later in life, indicating distinct timelines (6). The presence of genotype-specific ages of onset further supports divergent prognostic courses and highlights the need for age-tailored clinical management, including prognostic counseling and life planning.

To address these questions, we analyzed 506 patients spanning early adulthood to advanced age, all with clinically confirmed RP. We tested two hypotheses: first that the probability of detecting pathogenic variants with targeted resequencing differs with increasing age; and second that the age at symptom onset differs across causative genes, reflecting distinct trajectories among genetic subtypes.

## 2 Materials and methods

### 2.1 Study design and participants

We conducted a retrospective cross-sectional study using clinical and genetic data from the Kyushu University Hospital. Consecutive patients evaluated between 2002 and 2019 were included. The study protocol was approved by the Institutional Review Board of Kyushu University Hospital (Fukuoka, Japan), and patient’s informed consent was obtained in accordance with the Declaration of Helsinki. This study is an independent investigation focusing on the institutional patient population from Kyushu University (n = 506), which is distinct from the multicenter survey of 1,209 patients (7). While these 506 patients were included in the previous multicenter report (7), they represent the entire eligible population from our hospital at the time of this study for whom we held institutional ethical approval.

### 2.2 Inclusion and exclusion criteria

A clinical diagnosis of RP was confirmed by retinal specialists, as described previously (7). Diagnostic criteria included: (1) objective evidence of progressive photoreceptor and retinal pigment epithelial dysfunction; (2) characteristic imaging findings, including outer retinal atrophy on optical coherence tomography, abnormal autofluorescence, and bone spicule pigmentation on fundoscopy; (3) progressive peripheral field constriction; and (4) severely reduced or absent rod and cone responses on full-field electroretinography. We excluded patients with syndromic RP, cone-rod or cone dystrophy, Bietti crystalline retinopathy, uveitis, choroideraemia, Leber congenital amaurosis, or retinitis punctata albescens from the study. To reduce the effect of relatives, we also excluded patients other than the proband of each pedigree.

### 2.3 Genetic analysis

For genomic analyses, DNA samples were obtained from extracted peripheral blood. Targeted next-generation sequencing of 83 genes was performed on an Illumina platform, as described previously (7).

The complete list of targeted RP-associated genes is provided in **Supplementary Table 1**. This panel and the underlying sequencing data were identical to those used in our previous study (7). The sequencing technology and bioinformatics pipelines for variant detection remained unchanged to ensure data consistency. The present study incorporates and compares results from two interpretation frameworks—the initial 2019 criteria (7) and the updated 2024 criteria (8)—to demonstrate the robustness of the findings across different evaluative standards. Initially, variants were filtered and prioritized using the hierarchical classification system described previously (7). Subsequently, all variants underwent recuration according to the 2015 ACMG/AMP guidelines, following the protocol detailed in our recent study (8). ClinVar assertions without independent evaluation were not accepted. To maintain a conservative estimate of diagnostic yield, only pathogenic or likely pathogenic variants were included in the “solved” category, while all variants of uncertain significance (VUS) were excluded under the 2024 criteria.

### 2.4 Data collection and statistical analysis

Demographic and clinical variables, including age at symptom onset and age at enrollment, were retrospectively obtained from electronic health records. Genetic detection rates were calculated for six age groups (<40, 40–49, 50–59, 60–69, 70–79, and ≥80 years). The Cochran-Armitage test was used to assess trends in detection rates across age strata. Age at onset among genotype groups was compared using a one-way ANOVA with Tukey–Kramer *post hoc* tests. For small sample groups, a non-parametric Kruskal–Wallis test was also performed to confirm consistency. All analyses were conducted in R version 4.3.2, with the threshold of statistical significance defined as p < 0.05.

## 3 Results

### 3.1 Characteristics and genetic detection rate

**Figure 1:**
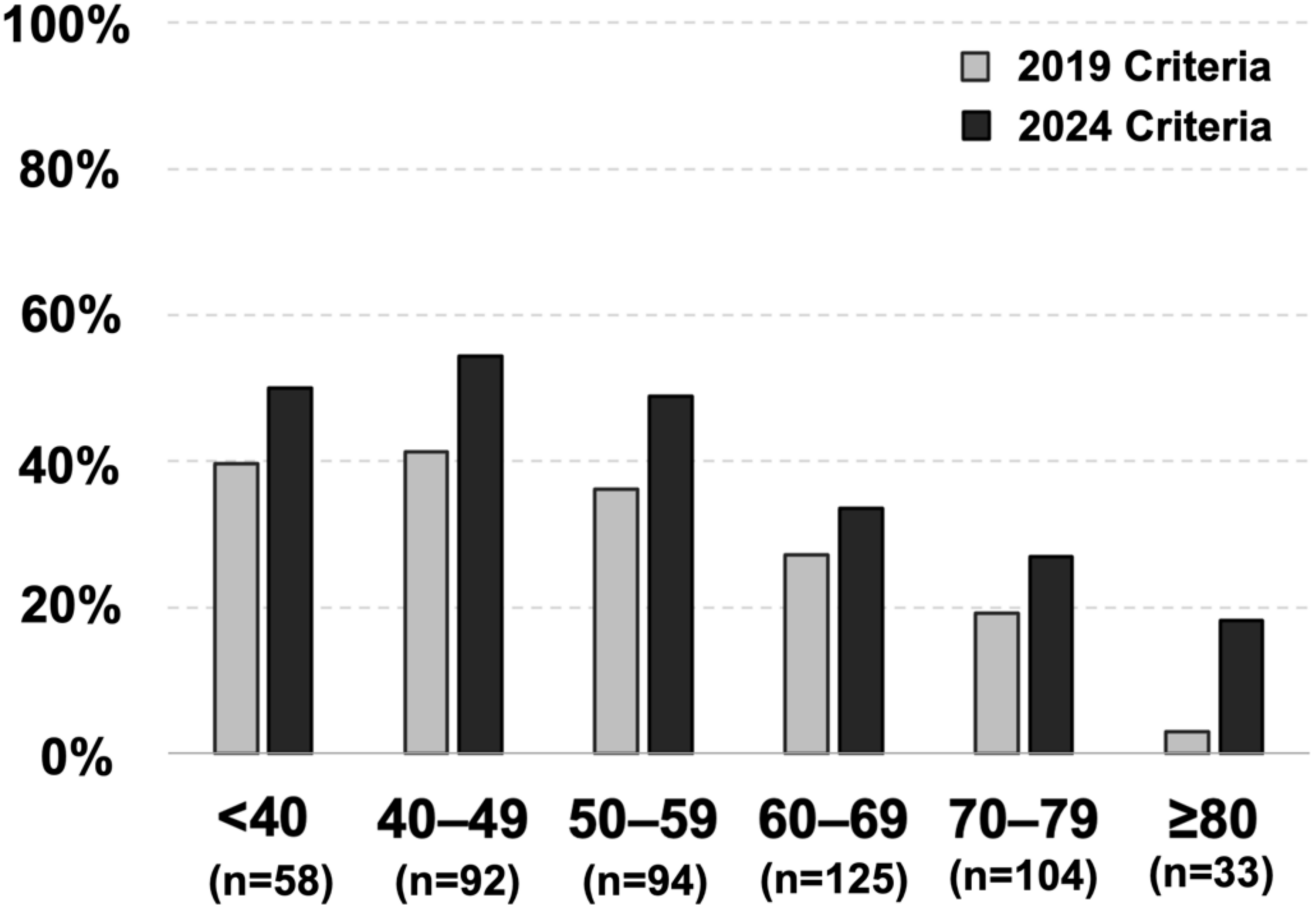
Detection rate of causative genes across age groups. This figure summarizes the diagnostic yield for each age group. The Cochran–Armitage trend test revealed a highly significant age-dependent decline in diagnostic yield based on the initial classification (p = 8.22 × 10^-7^). While the application of ACMG standards refined the specific results, the primary finding—the significant age-dependent decline in diagnostic yield—remained unchanged and statistically robust (Cochran–Armitage trend test: p = 3.85 × 10^-7^ for the 2024 Criteria). Detailed comparative data, including Diagnostic Yield (2019) [95% CI] and Diagnostic Yield (updated 2024) [95% CI], are provided in **Supplementary Table 2**.

In total, 506 patients satisfied the inclusion criteria. The mean age at enrollment was 58.8 ± 15.2 years (range, 23–98), and 235 (46.4%) were male. The overall genetic detection rate based on the previous 2019 criteria was 29.6% (i.e., 150 of 506). By age group, detection rates were 39.7% (<40), 41.3% (40–49), 36.2% (50–59), 27.2% (60–69), 19.2% (70–79), and 3.0% (≥80) under the 2019 criteria (**Figure 1**; **Supplementary Table 2**). The diagnostic yield differed significantly across age strata, with detection rates starting at 39.7% in patients younger than 40 years and decreasing to 3.0% in those 80 years or older, showing a strong inverse association with increasing age (Cochran-Armitage test, p = 8.22 × 10^-7^). Based on the 2024 Criteria, among genetically solved cases, 51 unique pathogenic variants and 55 unique likely pathogenic variants were identified, with the *RP1* Alu insertion variant p.(Tyr1352Alafs*9) also treated as a causative variant for genetic diagnosis (8). The overall genetic detection rate based on the previous 2024 criteria was 39.7% (i.e., 201 of 506). By age group, detection rates were 50.0% (<40), 54.3% (40–49), 48.9% (50–59), 33.6% (60–69), 26.9% (70–79), and 18.2% (≥80) under 2024 criteria (**Figure 1**; **Supplementary Table 2**). The diagnostic yield still exhibited a significant age-dependent decline (Cochran–Armitage trend test, p = 3.85 × 10^-7^).

### 3.2 Causative gene and inheritance mode-specific age of onset

Among the 150 cases with a genetic diagnosis in accordance with the 2019 criteria, cases harboring the major causative genes (*EYS*, *USH2A*, *RP1*, *RHO*, and *RPGR*) were included for analysis to compare age at onset across genetic subgroups, while the distribution of missing values for each gene is detailed in **Table 1**. The mean age at symptom onset varied significantly by gene (*p* < 0.001, one-way ANOVA) (**Table 1**). The earliest onset occurred in patients with the *RPGR* group, with a mean age of 5.2 years (n = 5). In contrast, the *USH2A* group had the latest onset, with a mean age of 34.1 years (n = 8). Mean onset ages for the *EYS*, *RP1*, and *RHO* groups were 19.5 years (n = 26), 25.2 years (n = 5), and 24.3 years (n = 4), respectively. A subsequent analysis showed that onset in the *RPGR* group was significantly earlier than in the *USH2A* group (*p* = 0.004). Regarding the age-at-onset analysis of the 150 genetically solved cases under the 2019 criteria, symptom onset data were available for only 74 patients (15 AD, 54 AR, and 5 XL cases) and unavailable for the remaining 76 patients (25 AD, 48 AR, and 3 XL). The results revealed that the age at onset was earliest in the XL group (mean: 6.2 years) as compared to the AD and AR groups (15.7 and 21.9 years, respectively), with a significant difference between the XL and AR groups (p = 0.012). These results indicate that specific causative genes and inheritance patterns exert a substantial influence on the age of disease onset.

**Table 1:** Age at onset and clinical characteristics by causative genes.

Feature	*EYS* Group (n=26)	*USH2A* Group (n=8)	*RP1* Group (n=5)	*RHO* Group (n=4)	*RPGR* Group (n=5)
Mean Age of Onset (years)*	19.5	34.1	25.2	24.3	5.2
Gender (Male/Female)	12/14	4/4	2/3	2/2	5/0
Mode of Inheritance	Autosomal Recessive: 26	Autosomal Recessive: 8	Autosomal Dominant: 4	Autosomal Dominant: 3	X-linked: 5
			Autosomal Recessive: 1	Autosomal Recessive: 1	

Data showing age at onset across causative genes. Onset age differed significantly among genes (p < 0.001, one-way ANOVA). For pairwise comparisons, the Wilcoxon rank-sum test was applied with Bonferroni correction to adjust for multiple testing. *The number of patients contributing to the age-at-onset analysis was identical to the total number (n) indicated for each group in the column headers. Among the cases identified by the 2019 criteria, the age at onset was missing for the remaining individuals (*EYS*, 16/42; *USH2A*, 11/19; *RP1*, 4/9; *RHO*, 10/14; and *RPGR*, 2/7).

## 4 Discussion

This study demonstrates a statistically significant negative correlation between genetic diagnostic yield and patient age in RP, supporting the hypothesis that the disease etiology may shift with age. Moreover, the age of disease onset differed depending on the causative gene detected, with *RPGR* group being associated with an earlier disease onset.

Several potential explanations may account for this trend. First, the increased prevalence of phenocopies in older adults warrants consideration. Conditions such as autoimmune or other infectious retinal degenerations can resemble the clinical and morphological features of RP, raising the possibility that some cases without clear genetic findings are clinically misclassified. Second, the potential contribution of oligogenic or complex genetic factors to late-onset phenotypes warrants consideration. Although digenic or quasi-Mendelian inheritance patterns have been proposed as contributors to the clinical variability of RP (9, 10), our analysis of 356 genetically unsolved cases revealed that only 7.6% (27/356) harbored pathogenic variants in two or more distinct autosomal recessive RP-associated genes (**Supplementary Table 4**). Notably, the Cochran–Armitage trend test showed no significant age-dependent enrichment for these multiple carriers (p = 0.14), suggesting that the precipitous decline in diagnostic yield in late-onset cases is not simply attributable to a cumulative burden of known pathogenic variants within coding regions. Instead, the lower diagnostic yield observed in late-onset cases may reflect limitations of targeted panel sequencing, including the potential contribution of VUS, the inability to detect structural, deep intronic, or regulatory variants, and the increased likelihood of phenocopies or other complex genetic or technical factors in older patients. Third, some late-onset cases may be attributed to variants in genes that have not yet been discovered or are not included in our targeted resequencing (11). This limitation may be reflected in the consistent decrease in diagnostic yield observed. These findings suggest that clinicians should consider initiating genetic testing using broader genomic sequencing (e.g., whole exome sequencing or whole genome sequencing) rather than relying on targeted panels, particularly for late-onset RP patients, while carefully considering the risk of secondary findings. These results also highlight the importance of integrating genomics, epigenetics, and environmental factors in research frameworks, which can capture the evolving disease etiology across the lifespan and inform diagnostic and therapeutic strategies tailored to age and genetic profiles (6).

Additionally, we observed that the age of onset differs based on causative gene, with the *RPGR* gene suggesting an earlier onset of disease and being associated with a more severe phenotype (6). This finding warrants further exploration into the relationship between specific genetic factors and disease manifestation across different age groups. Understanding how genetic variations influence the severity and the age at onset could provide critical insights for the development of age-stratified diagnostic and therapeutic strategies for RP.

This study has several limitations. This analysis is inherently limited because of its retrospective cross-sectional design. Although this design is useful for identifying associations between factors, it fundamentally prevents us from establishing a cause-and-effect relationship. A longitudinal design would be required. The retrospective nature of the study also introduced potential recall bias, particularly for the age of symptom onset. This bias may be more pronounced in older patients due to the longer recall interval, whereas onset may be recalled more accurately for early, dramatic symptoms than for insidious late-onset presentations. Furthermore, these data were collected from a single tertiary referral center, introducing referral bias: younger patients with severe, classic phenotypes may be overrepresented, while older patients with milder or more complex presentations may be underrepresented or less likely to undergo genetic testing. Additionally, the small sample size for some genetic subtypes reduced statistical power to detect subtle genotype-phenotype correlations and led to wide confidence intervals for mean onset ages. Multicenter collaborations will be essential to overcome these limitations and provide robust analyses of rare subtypes. Nevertheless, to validate our current observations, we performed a sub-analysis of a previously reported multicenter study (**Supplementary Table 3**) (7). This supplemental analysis confirmed a consistent age-dependent decline in diagnostic yield within the larger, nationwide population, thereby supporting the robustness of our current findings. Moreover, our design did not allow systematic accounting for age-related confounders, such as systemic health and comorbidities, which may independently or interactively influence retinal health and complicate diagnostic interpretation (12). Lastly, because pediatric individuals were not the focus of this study, we cannot evaluate their diagnostic yield or the underlying genetic background in that age range. These limitations warrant careful consideration in the interpretation of the study’s results.

In conclusion, the detection rate of known causative genes of RP was lower in the elderly patients, potentially reflecting factors associated with a late-onset phenotype.

## Data Availability

The raw data supporting the conclusions of this article will be made available by the authors, without undue reservation.

## Supplementary material

Supplementary Table 1 List of 83 targeted genes associated with RP.

Supplementary Table 2 Comparison of diagnostic yield with 95% CI (2019 vs. 2024 criteria). The Clopper–Pearson exact method based on the binomial distribution was used to calculate the 95% confidence intervals (CIs).

Supplementary Table 3 Diagnostic yield by age group in the previous multicenter study. The Cochran–Armitage trend test yielded a significant result (p = 7.06 × 10^-6^), confirming a decline in diagnostic yield with advancing age.

Supplementary Table 4 Frequency of autosomal recessive RP-associated variant carriers among genetically unsolved patients (n = 356). The Cochran–Armitage trend test revealed no significant age-dependent enrichment for these multiple carriers (p = 0.14).
