# Supplementary material for: Effects of age on the genetic and clinical characteristics of retinitis pigmentosa
Source: Front Ophthalmol (Lausanne). 2026 Jun 26;6:1776570. doi: 10.3389/fopht.2026.1776570 (PMC13349749; doi:10.3389/fopht.2026.1776570)
Supplement: Supplementary Table 1 — List of 83 targeted genes associated with RP. [file DataSheet1.pdf]

Supplementary Table 1. List of 83 targeted genes associated with RP.

**AD**	*ADIPOR1, ARL3, CA4, CRX, FSCN2, GUCA1B, HK1, IMPDH1, KLHL7, PRPF3, PRPF31, PRPF4, PRPF6, PRPF8, PRPH2, RDH12, ROM1, RP9, SEMA4A, SNRNP200, SPP2, TOPORS*
**AD/AR**	*BEST1, NRL, NR2E3, RHO, RP1, RPE65*
**AR**	*ABCA4, AGBL5, ARL2BP, ARL6, BBS1, BBS2, C2orf71, C8orf37, CERKL, CLRN1, CNGA1, CNGB1, CRB1, CYP4V2, DHDDS, DHX38, EMC1, EYS, FAM161A, GPR125, HGSNAT, IDH3B, IFT140, IFT172, IMPG2, KIAA1549, KIZ, LRAT, MAK, MERTK, MVK, NEK2, NEUROD1, PDE6A, PDE6B, PDE6G, POMGNT1, PRCD, PROM1, RBP3, RGR, RLBP1, RP1L1, SAG, SLC7A14, SPATA7, TRNT1, TTC8, TULP1, USH2A, ZNF408, ZNF513*
**XL**	*OFD1, RP2, RPGR*

Abbreviations: RP, retinitis pigmentosa; AD, autosomal dominant; AR, autosomal recessive; XL, X-linked.

Supplementary Table 2. Comparison of Diagnostic Yield with 95% CI (2019 vs. 2024 Criteria).

Age group (years)	Number of Patients (*n*)	Diagnostic Yield (2019) [95% CI]	Diagnostic Yield (Updated 2024) [95% CI]
<40	58	39.7% [27.0–53.4]	50.0% [36.6–63.4]
40–49	92	41.3% [31.1–52.1]	54.3% [43.6–64.7]
50–59	94	36.2% [26.5–46.7]	48.9% [38.5–59.5]
60–69	125	27.2% [19.6–35.9]	33.6% [25.4–42.6]
70–79	104	19.2% [12.2–28.1]	26.9% [18.6–36.6]
≥80	33	3.0% [0.1–15.8]	18.2% [7.0–35.5]

The Clopper–Pearson exact method based on the binomial distribution was used to calculate the 95% confidence intervals (CIs).

Supplementary Table 3. Diagnostic yield by age group in the previous multicenter study.

Age group (years)	Number of Patients (n)	Solved (2019 criteria)	Diagnostic Yield (2019 criteria) (%) [95% CI]
<40	253	88	34.8 [29.0–41.0]
40–49	230	83	36.1 [29.9–42.7]
50–59	224	70	31.3 [25.3–37.7]
60–69	298	78	26.2 [21.3–31.5]
70–79	163	34	20.9 [14.9–28.0]
≥80	36	3	8.3 [1.8–22.5]

The Cochran–Armitage trend test yielded a significant result ($p = 7.06 \times 10^{-6}$), confirming a decline in diagnostic yield with advancing age.

Supplementary Table 4. Frequency of autosomal recessive RP-associated variant carriers among genetically unsolved patients (n = 356).

Age at Testing (years)	Unsolved Cases (n)	Single AR-variant carrier (n,%)	Multiple AR-variant carriers (≥2 genes) (n,%)	Multiple AR-variant carriers (≥2 genes) (n,%)
<40	57	27	5	5 (18.5%)
40–49	56	36	7	7 (19.4%)
50–59	72	30	6	6 (20%)
60–69	102	48	5	5 (10.4%)
70–79	56	22	4	4 (18.2%)
≥80	13	6	0	0 (0%)

The Cochran–Armitage trend test revealed no significant age-dependent enrichment for these multiple carriers ($p = 0.14$).
